# Motor performance and its association with Alzheimer’s-related biomarkers: a systematic review

**DOI:** 10.1007/s10072-026-09253-4

**Published:** 2026-07-31

**Authors:** María Fernanda Serna, Mildrey Mosquera, Herney Andrés García-Perdomo

**Affiliations:** 1 Departamento de Ciencias Fisiológicas, Grupo de Nutrición, Facultad de Salud, Universidad del Valle, Cali, Colombia; 2 Faculty of Health, Universidad Santiago de Cali, Cali, Colombia; 3 Division of Urology/Urooncology, Department of Surgery, School of Medicine, Universidad del Valle, Cll 4b #36-00, Cali, Colombia

**Keywords:** Alzheimer disease, Mild cognitive impairment, Biomarkers, Motor activity, Neurodegenerative diseases

## Abstract

**Purpose:**

To examine associations between neurodegenerative and inflammatory biomarkers and motor function in adults with Alzheimer’s disease (AD), mild cognitive impairment (MCI), or at risk of AD.

**Methods:**

A systematic review was conducted using MEDLINE, Web of Science, Scopus, LILACS, and CENTRAL.

**Results:**

Seventeen studies (2018–2026) were included. CSF Aβ42 levels were associated with gait speed, balance, and mobility outcomes, while p-tau, p-tau181, p-tau217, and t-tau correlated with mobility impairments, dual-task performance, and reduced physical function. Higher neurofilament light chain (NfL) levels were linked to poorer Short Physical Performance Battery (SPPB) performance, slower gait speed, and lower grip strength. One study found serum IL-8 associated with mobility outcomes.

**Conclusions:**

AD-related biomarkers, particularly Aβ42, p-tau, p-tau181, p-tau217, t-tau, and NfL, show consistent associations with gait disturbances, mobility decline, and reduced physical function. Motor changes may precede cognitive decline, supporting their potential as early indicators of neurodegeneration.

**Supplementary Information:**

The online version contains supplementary material available at 10.1007/s10072-026-09253-4.

## Introduction

Alzheimer’s disease (AD) is one of the most prevalent forms of dementia [1, 2], characterized by progressive cognitive decline with mild cognitive impairment (MCI) representing an intermediate stage of the disease [1]. AD is marked by the accumulation of beta-amyloid (Aβ) peptide plaques, neurofibrillary tangles of tau protein, and an inflammatory response at both the central and peripheral levels [2]. Several studies have shown that plasma concentrations of these biomarkers change throughout different stages of the disease, and some have been investigated as potential markers for identifying populations at risk for AD [3]. Neurodegenerative and inflammatory biomarkers have been associated with cognitive decline, memory impairment, disorientation, and deficits in learning and executive function. However, growing evidence suggests that motor impairment may precede cognitive decline and progress alongside the disease continuum, making motor dysfunction a potential early indicator of AD-related neurodegeneration [4].

Evidence indicates that the most common motor impairments in AD patients are gait disturbances [5]. However, disruptions in balance, muscle atrophy, loss of dexterity, strength decline, and reduced cardiorespiratory fitness have also been reported [6]. These motor impairments contribute to physical dysfunction, restricting movement and limiting physical activities, thereby increasing the risk of falls and functional dependence. Motor and cognitive functions are closely linked, as poorer cognitive performance is associated with worse motor test outcomes, particularly in dual-task activities [7]. Dual-task gait assessments are valuable for evaluating motor capacity and predicting cognitive impairment [8].

Motor deficits in AD are most noticeable in the intermediate and late stages [9]; however, emerging evidence suggests they may also appear in the early stages and are associated with neuronal loss due to amyloid-β accumulation, tau pathology, neuroaxonal injury, and disruptions in motor pathways [10]. Brain inflammation plays a key role in the pathogenesis of neurodegenerative diseases, with elevated inflammatory marker profiles linked to a higher risk of dementia onset. High concentrations of pro-inflammatory cytokines have been associated with poorer cognitive and motor performance [11]. Although several studies have investigated associations between AD-related biomarkers measured in cerebrospinal fluid and blood and cognitive outcomes [12], evidence regarding their relationship with motor function remains fragmented across populations with AD, MCI, motoric cognitive risk syndrome (MCR), cognitive complaints, and cognitively unimpaired older adults at risk of dementia. Therefore, we aimed to examine the associations between neurodegenerative and inflammatory biomarkers and motor function in adults with Alzheimer’s disease, mild cognitive impairment, or at risk of Alzheimer’s disease.

### Methods

This review was conducted following the recommendations of the Cochrane Collaboration and following the PRISMA Statement.

### Eligibility criteria

We included cross-sectional, cohort, and case-control studies that met the following criteria: participants diagnosed with Alzheimer’s disease (AD), mild cognitive impairment (MCI), motoric cognitive risk syndrome (MCR), cognitive complaints, or cognitively unimpaired adults at risk of AD; assessment of at least one motor function outcome; and measurement of neurodegenerative or inflammatory biomarkers in blood or cerebrospinal fluid. Eligible studies reported either associations between biomarker levels and motor outcomes or comparisons of biomarker concentrations according to motor performance. Longitudinal studies evaluating AD-related biomarkers and motor function trajectories were also included.

Exclusion criteria comprised studies conducted in animal models or cell lines, populations with neurodegenerative disorders other than AD, and studies that did not report motor outcomes or biomarkers relevant to the review objectives. When studies included multiple diagnostic groups, only data from the populations of interest were extracted.

### Information sources

The literature search used Medical Subject Headings (MeSH), DeCS, and related text words. Searches were conducted in MEDLINE (OVID), Web of Science, Scopus, LILACS, and the Cochrane Central Register of Controlled Trials (CENTRAL) from inception through June 2026. To ensure literature saturation, the reference lists of relevant articles, thesis databases, OpenGrey, and Google Scholar were also screened. No language or publication date restrictions were applied. The complete search strategy for each database is provided in Appendix 1.

### Study selection

Two researchers independently performed double-masked review of the articles titles and abstracts to determine their potential usefulness. Eligibility criteria were applied during the full-text review of potentially eligible articles. They resolved any discrepancies through discussion and consensus. In cases without consensus, a third reviewer made the final decision.

### Data collection

Two researchers independently extracted data using a standardized form that included study design, publication year, country, sample size, diagnostic groups, age, sex, biomarker concentrations, motor outcomes, cognitive measures, and main findings. When available, biomarker concentrations and motor outcomes were extracted as means ± standard deviations or medians with interquartile ranges, according to the original study reports. Biomarker concentrations were converted to a common unit of measurement (pg/mL) whenever possible, except for C-reactive protein, which was reported in its original units. All extracted data were independently verified by both reviewers to ensure accuracy and consistency.

### Risk of bias and methodological quality assessment

Methodological quality was independently assessed by two reviewers using the Newcastle–Ottawa Scale (NOS) for cohort and case-control studies and an adapted version for cross-sectional studies, following Cochrane Collaboration recommendations. The NOS evaluates three domains: selection, comparability, and outcome/exposure, with a total score ranging from 0 to 9 points. Discrepancies between reviewers were resolved through discussion and consensus, with a third reviewer consulted when necessary. Studies were classified as low quality (0–3 points), moderate quality (4–6 points), and high quality (7–9 points) [13].

## Results

### Study selection

**Fig. 1:**
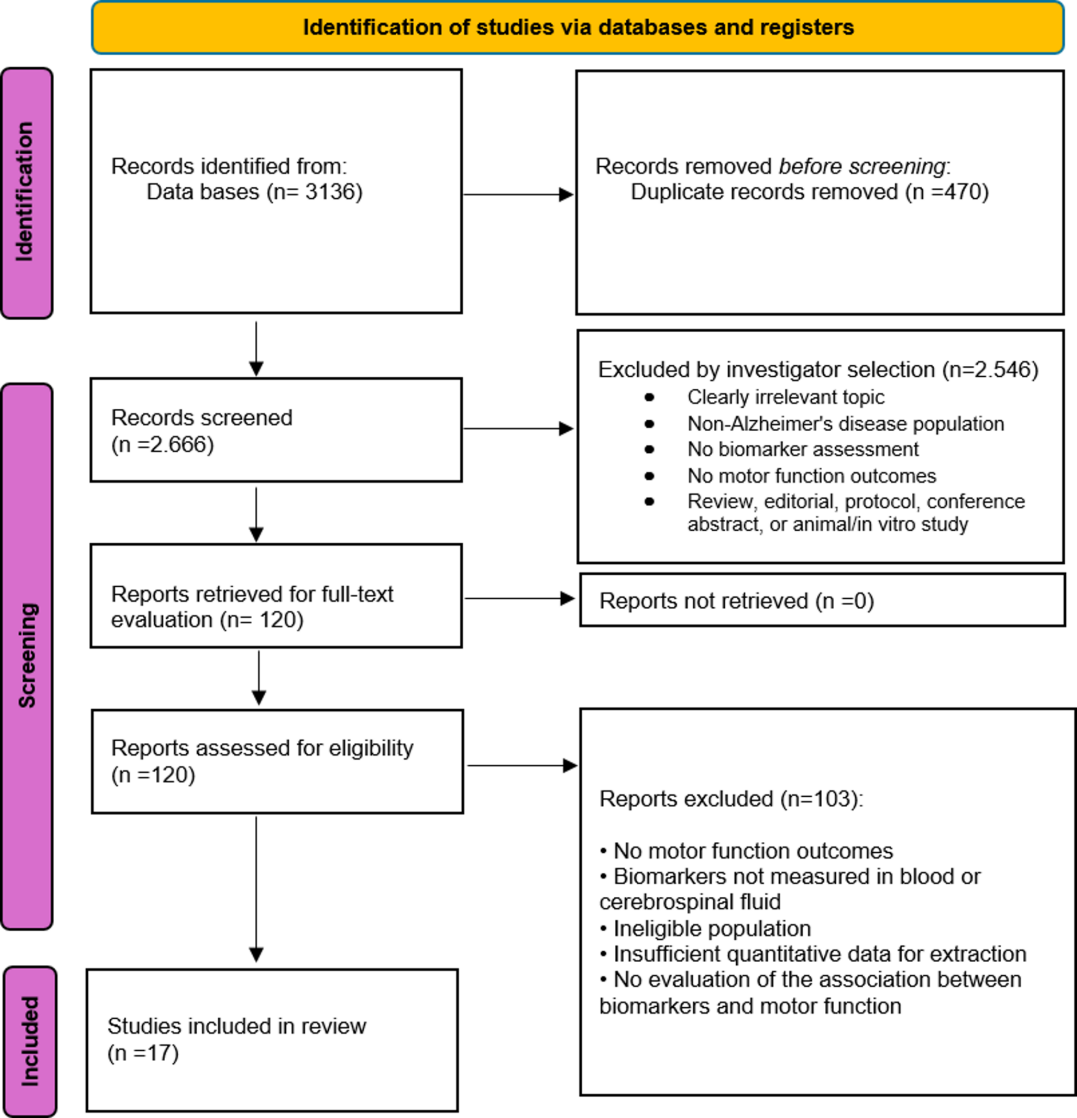
PRISMA flow diagram of the study selection process

A total of 3,136 records were identified through the database search. After removing 470 duplicate records, 2,666 records remained for title and abstract screening. Following investigator selection, 2,546 records were excluded, and 120 full-text articles were assessed for eligibility. Of these, 103 studies were excluded because they did not meet the predefined eligibility criteria, including the absence of motor function outcomes, lack of Alzheimer’s disease-related neurodegenerative or inflammatory biomarkers measured in blood or cerebrospinal fluid, inclusion of populations outside the scope of the review, insufficient quantitative data for extraction, or failure to evaluate the association between biomarkers and motor function. Finally, 17 studies [13–29] met the eligibility criteria and were included in the qualitative synthesis, comprising 10 cross-sectional and seven longitudinal studies (Fig. 1).

### Characteristics of excluded studies

Studies excluded during the full-text review did not meet the predefined eligibility criteria. The main reasons for exclusion were the absence of motor function outcomes, the lack of Alzheimer’s disease-related neurodegenerative or inflammatory biomarkers measured in blood or cerebrospinal fluid, inclusion of populations outside the scope of the review, insufficient quantitative data for data extraction (e.g., studies reporting only correlation or regression coefficients without biomarker concentrations or studies that did not report motor function results separately), and failure to evaluate the association between biomarkers and motor function. In addition, editorials, conference abstracts, systematic and narrative reviews, study protocols, pharmacological intervention studies, and animal studies were excluded.

### Characteristics of included studies

The 17 studies included in this review were published between 2018 and 2026 and comprised 10 cross-sectional studies [14, 17, 18, 20–23, 26, 28, 29] and seven prospective cohort studies [13, 15, 16, 19, 24, 25, 27]. Follow-up periods in the longitudinal studies ranged from 2 years [25] to 15 years [27]. Among the included studies, two studies [14, 20] enrolled participants with AD and MCI, three [18, 23, 26] included individuals with MCI and cognitively normal controls, and four studies [13, 16, 22, 24] focused on adults at risk of AD or those presenting cognitive complaints. Three studies [15, 17, 19] investigated cognitively unimpaired older adults, one study [25] included participants with AD, MCI, and cognitively normal controls, and two studies [21, 29] included individuals with motoric cognitive risk syndrome (MCR) in addition to AD and/or cognitively normal groups. The most recent longitudinal study [27] evaluated trajectories of motor and cognitive decline in a population-based cohort over a 15-year follow-up period (Tables 1 and 2).

**Table 1:** Characteristics and main findings of cross-sectional studies investigating associations between Alzheimer’s disease biomarkers and motor performance

Study	Objective	*n*	Population and subgroups	Sex (% female)	Age (years)	Clinical diagnostic criteria	Main findings
Åhman et al., 2019 [20]	To investigate correlations between Timed Up-and-Go dual-task (TUGdt) outcomes and Alzheimer’s disease cerebrospinal fluid (CSF) biomarkers Aβ42, total tau (t-tau), andphosphorylated tau (p-tau).	90	AD/MCI	42	49–84	NR	Performance during TUG dual-task with animal naming was negatively correlated with CSF t-tau and p-tau levels. No associations were found between completion time and CSF biomarkers.
Legdeur et al., 2020 [23]	To determine the association of risk factors, white matter hyperintensity volume, hippocampal atrophy, and amyloid aggregation with cognition in the oldest-old.	122	84 NC / 38 cognitively impaired	NC: 53.6; CI: 68.4	NC: 92.8 ± 2.9; CI: 91.6 ± 2.4	AD: NINCDS-ADRDA MCI: Petersen criteria NC: CDR = 0	Lower handgrip strength and SPPB scores were associated with worse cognition. Higher WMH volume and amyloid burden were associated with poorer cognitive performance. No significant association was observed between CRP levels and cognition.
Nilsson et al., 2020 [18]	To investigate whether white matter lesions, amyloid pathology, and tau pathology are independently associated with mobility, dual-tasking, and dynamic balance performance.	299	175 NC / 124 MCI	NC: 49.1; MCI: 45.2	NC: 72.5 ± 5.6; MCI: 70.9 ± 5.4	MCI: NIA-AA NC: MMSE 28–30	Higher CSF p-tau was associated with worse dual-task performance (TUG-Cog and dual-task cost). Lower CSF Aβ42/40 ratio was associated with poorer dynamic balance.
Tsai et al., 2021 [22]	To compare the effects of family history of AD and ApoE ε4 genotype on molecular biomarkers and neurocognitive performance.	44	Healthy ApoE ε4 carriers / Healthy non-ApoE ε4 carriers	50	ApoE ε4: 57.3 ± 7.3; Non-ApoE ε4: 59.7 ± 5.7	APOE ε4 genotype	IL-8 levels correlated with 8-Foot Up-and-Go performance. BDNF correlated with estimated VO₂max. Aβ1–40 and Aβ1–42 levels were associated with cardiorespiratory fitness and muscular performance.
Chen et al., 2022 [21]	To determine plasma Aβ42 and total tau levels and their relationships with cognition in individuals with motoric cognitive risk syndrome (MCR).	68	25 NC / 27 MCR / 16 AD	NC: 36.0; MCR: 51.9; AD: 81.2	NC: 74.3 ± 7.4; MCR: 75.2 ± 6.4; AD: 78.2 ± 6.6	AD: NIA-AA MCR: Verghese et al. (2014) criteria NC: CDR = 0	Plasma t-tau levels were significantly higher in MCR and AD compared with NC. Plasma t-tau was associated with MoCA and Boston Naming Test scores in the MCR group.
O’Bryant et al., 2023 [17]	To examine the relationship between plasma AD biomarkers and physical functioning outcomes among cognitively unimpaired adults.	1328	Cognitively unimpaired adults	64	66.1 ± 8.5	CDR = 0	Plasma Aβ40, Aβ42, and NfL were associated with TUG performance. Plasma Aβ40, Aβ42, t-tau, and NfL were associated with SPPB total score.
Sampatakakis et al., 2023 [26]	To investigate the relationship between physical function and CSF biomarkers across the Alzheimer’s disease continuum.	163	112 NC / 51 MCI	NC: 67.9; MCI: 70.6	NC: 62.8 ± 9.2; MCI: 67.6 ± 8.2	MCI: Petersen criteria NC: NR	Walking time was inversely associated with CSF Aβ42, particularly in MCI and participants older than 60 years. Handgrip strength was negatively associated with CSF tau and p-tau in males.
Tangen et al., 2023 [14]	To explore associations between mobility measures and CSF core AD biomarkers.	64	15 MCI / 49 AD	AD: 44.9; MCI: 45.3	MCI: 67.7 ± 6.3; AD: 69.1 ± 7.4	AD: NIA-AA MCI: NIA-AA	Higher CSF Aβ42 was associated with better Mini-BESTest performance, faster gait speed, lower TUG time, and lower TUG dual-task cost. T-tau was associated with TUG dual-task cost, whereas p-tau181 showed no significant associations.
Thompson et al., 2025 [29]	To examine associations between plasma biomarkers of Alzheimer’s disease and mobility performance in cognitively unimpaired older adults.	192	Cognitively unimpaired older adults	NR	≥ 70 years	MoCA scores MCI < 20	Higher plasma p-tau181 was associated with worse eSPPB performance, slower gait speed, and poorer balance. Higher NfL was associated with worse eSPPB performance and slower gait speed. No associations were observed for Aβ42/40, p-tau217, or GFAP.
Chen et al., 2025 [28]	To examine the cross-sectional associations between plasma Aβ42 and total tau levels and physical function in older adults with normal cognition, motoric cognitive risk syndrome, and mild Alzheimer’s disease.	159	52 NC / 62 MCR / 45 AD	NC: 53.8; MCR: 59.7; AD: 57.8	NC: 73.9 ± 7.1; MCR: 74.8 ± 7.1; AD: 79.6 ± 6.9	AD: NIA-AA MCR: Verghese et al. (2014) criteria NC: MMSE	Higher plasma Aβ42 was associated with poorer TUG performance, particularly in MCR. Higher plasma total tau was positively associated with gait speed in MCR. No associations were observed with balance or dual-task performance.

*AD* Alzheimer’s disease, *MCI* mild cognitive impairment, *NC* normal cognition, *MCR* motoric cognitive risk syndrome, *CSF* cerebrospinal fluid, *NR* not reported, *Aβ42* amyloid-beta 42, *Aβ42/40* amyloid-beta 42/40 ratio, *t-tau* total tau, *p-tau* phosphorylated tau, *NfL* neurofilament light chain, *GFAP* glial fibrillary acidic protein, *CRP* C-reactive protein, *SPPB* Short Physical Performance Battery, *TUG* Timed Up and Go, *TUG-Cog* Timed Up and Go with cognitive task, *Mini-BESTest* Mini Balance Evaluation Systems Test, *BDNF* brain-derived neurotrophic factor, *VO*₂*max* maximal oxygen consumption, *WMH* white matter hyperintensity

**Table 2:** Characteristics and main findings of longitudinal studies investigating associations between Alzheimer’s disease-related biomarkers and motor function outcomes

Authors	Objective	Follow-up time (years)	Population and subgroups	*n*	Sex (% female)	Age (years)	Clinical diagnostic criteria	Main findings
Skillbäck et al., 2021 [19]	To examine the risk of cognitive decline in community-dwelling subjects with decreased gait speed but intact cognition.	15	Healthy older adults at risk	287	57.1–57.5	Baseline: 79; Follow-up: 85	CDR = 0	Aβ-positive participants experienced a significantly greater decline in gait speed compared with Aβ-negative participants. Baseline gait speed predicted future cognitive decline.
He et al., 2020 [16]	To investigate cross-sectional and longitudinal associations between plasma neurodegenerative biomarkers and physical performance.	5	Community-dwelling older adults (MAPT cohort)	507	NR	76 ± 5	MMSE > 24	Higher plasma NfL concentrations were associated with lower SPPB scores in cross-sectional analyses. Higher PGRN levels were associated with a greater decline in handgrip strength over time. No significant associations were observed for Aβ42/Aβ40 ratio.
Paulsen et al., 2022 [13]	To characterize 10-year changes in blood biomarkers of neurodegeneration and Alzheimer’s disease pathology and determine whether sensory and motor function are associated with biomarker trajectories.	10	Healthy middle-aged and older adults	1529	54.2	49.2 ± 9.4	NR	Poorer motor performance was associated with faster increases in serum NfL concentrations over time, particularly among women. Lower baseline grip strength was associated with higher baseline NfL levels.
Grasset et al., 2024 [24]	To investigate relationships between baseline blood-based Alzheimer’s disease biomarkers and trajectories of cognitive and functional decline in the MEMENTO cohort.	5	Adults with cognitive complaints, dementia-free at baseline	1938	62.0	72.8 ± 6.6	CDR ≤ 0.5	Higher baseline p-tau181 and NfL concentrations were associated with faster decline in SPPB performance and functional abilities. Lower Aβ42/40 ratio was associated with faster cognitive decline.
Jacob et al., 2022 [15]	To examine associations between physical function measures and plasma biomarkers of Alzheimer’s disease and neurodegeneration.	10	Dementia-free middle-aged and older adults (Framingham Heart Study)	2336	54.3	61.0 ± 9.0	NR	Grip strength was associated with plasma Aβ40 and total tau concentrations. Faster gait speed was associated with lower plasma total tau levels. No significant associations were found for Aβ42.
Nielsen et al., 2018 [25]	To investigate the diagnostic and prognostic ability of a dual-task paradigm in patients with MCI and evaluate associations with cerebrospinal fluid Alzheimer’s disease biomarkers.	2	41 NC / 29 MCI / 17 AD	86	NC: 47.1; MCI: 33.3; AD: 19.5	NC: 66.2 ± 7.3; MCI: 72.0 ± 8.6; AD: 69.0 ± 8.9	MMSE > 22	Dual-task cost was positively correlated with CSF t-tau and p-tau levels and negatively correlated with CSF Aβ42 levels and the Aβ42/p-tau ratio, suggesting that dual-task impairment reflects underlying Alzheimer’s disease pathology.
Pinardi et al., 2026 [27]	To investigate the association between blood-based Alzheimer’s disease biomarkers and patterns of cognitive and motor decline over 15 years.	15	Dementia-free community-dwelling older adults	1660	62.4	71.0 ± 9.8	NR	Higher p-tau217, p-tau181, and NfL levels were strongly associated with dual cognitive-motor decline. Lower Aβ42/40 ratio and higher GFAP concentrations were mainly associated with cognitive decline, whereas p-tau217 was also associated with isolated motor decline.

*AD* Alzheimer’s disease, *NC* normal cognition; MCI, mild cognitive impairment, *Aβ42* amyloid-beta 42, *Aβ42/40* amyloid-beta 42/40 ratio, *t-tau* total tau, *p-tau181* phosphorylated tau 181, *p-tau217* phosphorylated tau 217, *NfL* neurofilament light chain, *GFAP* glial fibrillary acidic protein, *PGRN* progranulin, *SPPB* Short Physical Performance Battery, *CSF *cerebrospinal fluid

All studies included both male and female participants, with sample sizes ranging from 44 [22] to 2860 [15]. Most studies reported a higher proportion of women among participants with cognitive impairment or AD. Ten studies aimed to investigate associations between AD-related biomarkers and motor outcomes, including gait, mobility, balance, strength, and physical performance [14–18, 20, 21, 25, 26, 29], whereas seven studies primarily evaluated the relationship between biomarkers, cognitive decline, and longitudinal changes in motor function [13, 19, 22–24, 27].

### Risk of bias assessment

The methodological quality assessment using the Newcastle–Ottawa Scale (NOS) is presented in Tables S1 and S2. Overall, 12 studies were classified as high quality and 5 as moderate quality, with no studies rated as low quality. The seven longitudinal studies achieved NOS scores ranging from 6 to 8 points, with the most common sources of potential bias related to the absence of non-exposed cohorts and limitations in follow-up assessment. The ten cross-sectional studies obtained scores ranging from 6 to 9 points, with the main methodological limitations associated with sample representativeness, participant selection, and adjustment for potential confounding factors. Despite these limitations, the overall methodological quality of the included studies was considered satisfactory.

### Biomarkers of Alzheimer’s disease

Tables 3 and 4 summarize the biomarker findings reported in the included studies. Neurodegenerative biomarkers were assessed in 16 of the 17 studies [13–22, 24–26] whereas inflammatory biomarkers were evaluated in three studies [16, 22, 23]. Regarding biological samples, six studies [14, 18–20, 25, 26] measured biomarkers in cerebrospinal fluid, seven studies [15–17, 21, 27–29] used plasma samples, and four studies [13, 22–24] analyzed serum samples.

**Table 3:** Associations between Alzheimer’s disease-related biomarkers, motor function, and cognitive function in cross-sectional studies

Study	Type of sample	Biomarker	Biomarker concentration	*p*-value biomarker	Type of motor assessment	Motor function measure	Result of motor measure	*p*-value	Cognitive assessment	Result of cognitive function
Tangen et al., 2023 [14]	CSF	Aβ42	MCI: 881.6 ± 306.6 / AD: 626.1 ± 197.5	< 0.001	Balance	Mini-BESTest (score)	MCI: 24.7 ± 3.0 / AD: 22.2 ± 3.5	0.021	MMSE	MCI: 29.0 ± 1.0 / AD: 25.0 ± 6.0
		p-tau181	MCI: 69.9 ± 33.0 / AD: 85.5 ± 32.0	0.100	Mobility	TUG simple (s)	MCI: 9.9 ± 2.2 / AD: 11.1 ± 2.7	0.140		
		t-tau	MCI: 438.4 ± 297.9 / AD: 659.3 ± 284.7	0.012	Mobility	TUG dual-task cost (%)	MCI: −0.7 ± 14.8 / AD: −21.8 ± 33.4	< 0.001		
					Gait	Gait speed (m/s)	MCI: 1.04 ± 0.16 / AD: 1.01 ± 0.19	0.620		
Nilsson et al., 2020 [18]	CSF	p-tau	MCI: 64.7 ± 26.6 / NC: 54.5 ± 20.6	NR	Mobility	TUG simple (s)	MCI: 12.4 ± 4.9 / NC: 11.0 ± 2.8	NR	MMSE	MCI: 26.8 ± 1.8 / NC: 28.7 ± 1.3
					Mobility	TUG dual-task (s)	MCI: 27.2 ± 26.0 / NC: 18.3 ± 10.6	NR		
Åhman et al., 2019 [20]	CSF	Aβ42	429.53 ± 180.75	NR	Mobility	TUG simple (s)	12.7 ± 3.1	NR	MMSE	25 ± 3.03
		t-tau	380.5 ± 247.68	NR	Mobility	TUG dual-task naming animals (s)	14.3 ± 4.16	NR		
		p-tau	46.4 ± 26.7	NR	Mobility	TUG dual-task months backward (s)	16.3 ± 5.97	NR		
Chen et al., 2022 [21]	Plasma	Aβ42	AD: 16.8 ± 0.5 / MCR: 16.6 ± 0.6 / NC: 16.4 ± 0.5	0.106	Gait	Gait speed (m/s)	AD: 0.8 ± 0.3 / NC: 1.1 ± 0.2	< 0.001	MMSE	AD: 15.3 ± 5.4 / MCR: 23.7 ± 4.6 / NC: 26.6 ± 2.8
		t-tau	AD: 24.2 ± 3.6 / MCR: 22.7 ± 3.4 / NC: 20.6 ± 1.7	< 0.001						
Tsai et al., 2021 [22]	Serum	TNF-α	Non-ApoE-4: 2.18 ± 0.65 / ApoE-4: 2.31 ± 0.62	0.629	Strength	Handgrip (kg)	Non-ApoE-4: 27.32 ± 5.68 / ApoE-4: 30.68 ± 10.09	0.446	MMSE	Non-ApoE-4: 29.32 ± 1.21 / ApoE-4: 29.23 ± 1.07
		IL-1β	Non-ApoE-4: 0.21 ± 0.10 / ApoE-4: 0.19 ± 0.08	0.604	Strength	Arm Curl (repetitions)	Non-ApoE-4: 22.36 ± 6.84 / ApoE-4: 26.68 ± 8.78	0.055		
		IL-6	Non-ApoE-4: 0.32 ± 0.50 / ApoE-4: 0.46 ± 0.79	0.265	Strength	30-s Chair Stand (repetitions)	Non-ApoE-4: 18.36 ± 7.15 / ApoE-4: 19.09 ± 5.58	0.390		
		IL-8	Non-ApoE-4: 2.39 ± 1.37 / ApoE-4: 2.18 ± 1.60	0.250	Mobility	8-Foot Up-and-Go (s)	Non-ApoE-4: 5.28 ± 1.01 / ApoE-4: 5.20 ± 0.58	0.857		
		Aβ1–40	Non-ApoE-4: 210.49 ± 285.41 / ApoE-4: 474.34 ± 1218.64	0.299	Cardiorespiratory	VO₂max (mL/kg/min)	Non-ApoE-4: 31.12 ± 4.24 / ApoE-4: 27.83 ± 6.50	0.083		
		Aβ1–42	Non-ApoE-4: 28.82 ± 35.34 / ApoE-4: 30.96 ± 38.63	0.848						
Legdeur et al., 2020 [23]	Serum	CRP (mg/L)	MCI: 3.2 ± 3.5 / NC: 2.9 ± 2.9	0.850	Strength	Handgrip males (kg)	MCI: 17.5 ± 6.8 / NC: 22.5 ± 6.7	0.040	MMSE	MCI: 23.8 ± 3.3 / NC: 28.5 ± 1.5
					Strength	Handgrip females (kg)	MCI: 9.9 ± 5.4 / NC: 12.3 ± 4.0	0.080		
					Physical function	SPPB (points)	MCI: 6.2 ± 2.6 / NC: 8.0 ± 2.7	< 0.010		
O’Bryant et al., 2023 [17]	Plasma	Aβ40	250.81 ± 65.93	< 0.001	Mobility	TUG (s)	9.66 ± 2.30	< 0.001	MMSE	28.07 ± 2.30
		Aβ42	11.99 ± 3.25	0.014	Physical function	SPPB (score)	10.88 ± 1.82	< 0.001		
		t-tau	2.44 ± 0.93	< 0.001						
		NfL	18.18 ± 11.37	< 0.001						
Sampatakakis et al., 2023 [26]	CSF	Aβ42	MCI: 989.2 ± 538.3 / NC: 1240.0 ± 508.5	0.005	Gait	Walking time (s)	MCI: 5.82 ± 2.65 / NC: 4.33 ± 1.04	< 0.001	MMSE	MCI: 26.4 ± 1.8 / NC: 28.9 ± 1.2
		t-tau	MCI: 257.9 ± 96.5 / NC: 218.0 ± 196.9	0.171	Strength	Handgrip (kg)	MCI: 23.3 ± 7.8 / NC: 26.8 ± 9.3	0.032		
		p-tau	MCI: 23.6 ± 10.7 / NC: 17.2 ± 8.9	< 0.001						
Thompson et al., 2025 [29]	Plasma	p-tau181	NR	Significant	Physical function	eSPPB (score)	Higher p-tau181 associated with lower eSPPB score	< 0.05	Global cognition	No significant association
		NfL	NR	Significant	Gait	4-m gait speed	Higher NfL associated with slower gait speed	< 0.05		
		NfL	NR	Significant	Gait	400-m gait speed	Higher NfL associated with slower gait speed	< 0.05		
		p-tau181	NR	Significant	Balance	Balance time	Higher p-tau181 associated with poorer balance	< 0.05		
Chen et al., 2025 [28]	Plasma	Aβ42	NR	Significant	Mobility	TUG (s)	Higher Aβ42 associated with poorer TUG performance	< 0.05	MMSE	AD < MCR < NC
		t-tau	NR	Significant	Gait	Gait speed (m/s)	Higher t-tau associated with gait speed in MCR	< 0.05		
		Aβ42	NR	NS	Balance	Tandem stance	No significant association	NS		
		Aβ42	NR	NS	Mobility	Dual-task walking	No significant association	NS		

*AD* Alzheimer’s disease, *NC* normal cognition, *MCI* mild cognitive impairment, *MCR* motoric cognitive risk syndrome, *CSF* cerebrospinal fluid, *TUG* Timed Up and Go, *TUG-DT* Timed Up and Go dual task, *SPPB* Short Physical Performance Battery, *eSPPB* expanded Short Physical Performance Battery, *MMSE* Mini-Mental State Examination, *CRP* C-reactive protein, *NfL* neurofilament light chain, *BDNF* brain-derived neurotrophic factor, *VO*₂*max* maximal oxygen consumption, *NR* not reported, *NS* not significant

**Table 4:** Associations between Alzheimer’s disease-related biomarkers, motor function, and cognitive function in longitudinal studies

Study	Type of sample	Biomarker	Biomarker concentration	*p*-value biomarker	Type of motor assessment	Motor function measure	Result of motor measure	*p*-value	Cognitive assessment	Result of cognitive function
Skillbäck et al., 2021 [20]	CSF	Aβ1–42	Baseline: 810 ± 216; Follow-up: 829 ± 223.1	0.003	Gait	Maximum gait speed (s/30 m)	Baseline: 21.2 ± 5.2; Follow-up: 22.1 ± 4.7	< 0.001	NR	NR
					Gait	Normal gait speed (s/30 m)	Baseline: 28.7 ± 5.4; Follow-up: 29.2 ± 6.0	< 0.001		
He et al., 2020 [16]	Plasma	Aβ42/Aβ40 ratio	0.11 ± 0.01	NR	Gait	Gait speed (m/s)	1.05 ± 0.24	NR	MMSE	28 ± 2
		NfL	84.06 ± 56.99	NR	Physical function	SPPB (score)	11 ± 2	NR		
		PGRN	45.43 ± 11.54	NR	Strength	Chair stands (stands/s)	11.3 ± 4.2	NR		
					Strength	Grip strength (kg)	26.6 ± 9.9	NR		
Paulsen et al., 2022 [13]	Serum	NfL	Baseline: 11.1 ± 7.6; Follow-up change: 4.6 ± 11.0	NR	Dexterity	Grooved Pegboard Test (s)	71.7 ± 15.6	NR	NR	NR
		Aβ42/40 ratio	Baseline: 0.077 ± 0.023; Follow-up change: −0.004 ± 0.022	NR	Strength	Grip strength (kg)	38.4 ± 12.4	NR		
		t-tau	Baseline: 0.71 ± 7.0; Follow-up change: 0.03 ± 0.79	NR						
Jacob et al., 2022 [15]	Plasma	Aβ40	158.17 ± 35.25	NR	Strength	Grip strength (kg)	33.05 ± 12.86	NR	NR	NR
		Aβ42	43.51 ± 9.95	NR	Strength	Chair stand speed (stands/s)	0.45 ± 0.14	NR		
		t-tau	3.91 ± 1.15	NR	Gait	Gait speed (m/s)	Baseline: 1.73 ± 0.45; Follow-up: 1.60 ± 0.38	NR		
Grasset et al., 2024 [24]	Serum	Aβ42/40 ratio	0.06 ± 0.02	NR	Physical function	SPPB (score)	10.5 ± 1.9	NR	MMSE	27.9 ± 1.9
		p-tau181	1.07 ± 0.80	NR			Higher p-tau181 associated with faster SPPB decline	Significant		
		NfL	21.7 ± 13.1	NR			Higher NfL associated with faster SPPB decline	Significant		
Nielsen et al., 2018 [25]	CSF	Aβ42	AD: 553.5 ± 222.7 / MCI: 752.3 ± 388.3 / NC: 965.3 ± 276.5	< 0.010	Mobility	TUG simple (s)	AD: 9.1 ± 3.15 / MCI: 8.3 ± 1.48 / NC: 7.7 ± 1.31	NS	MMSE	AD: 26.2 / MCI: 27.8 ± 1.95 / NC: 29.0 ± 1.56
		p-tau	AD: 81.3 ± 22.6 / MCI: 57.0 ± 27.3 / NC: 49.7 ± 20.7	< 0.010						
		t-tau	AD: 573 ± 501.5 / MCI: 389 ± 240.9 / NC: 289 ± 138.2	< 0.010	Mobility	TUG dual-task (s)	AD: 14.9 ± 8.08 / MCI: 10.7 ± 3.74 / NC: 8.3 ± 1.92	< 0.010		
		Aβ42/p-tau ratio	AD: 6.77 ± 4.2 / MCI: 14.77 ± 11.62 / NC: 21.33 ± 10.6	< 0.010						
Pinardi et al., 2026 [27]	Plasma	Aβ42/40	Slow/non-decliners: 0.06 [0.05–0.07]; Fast motor decliners: 0.06 [0.05–0.07]; Fast cognitive decliners: 0.05 [0.05–0.06]; Dual decliners: 0.05 [0.05–0.06]	< 0.001	Gait	Gait speed (m/s)	Slow/non-decliners: 1.25 ± 0.30; Fast motor decliners: 0.95 ± 0.38; Fast cognitive decliners: 1.04 ± 0.34; Dual decliners: 0.72 ± 0.40	< 0.001	MMSE	Slow/non-decliners: 29.35 ± 0.83; Fast motor decliners: 29.13 ± 0.94; Fast cognitive decliners: 27.94 ± 1.73; Dual decliners: 27.59 ± 2.38
		p-tau217	0.07 [0.04–0.12]; 0.11 [0.06–0.19]; 0.13 [0.07–0.22]; 0.18 [0.10–0.29]	< 0.001	Gait	Cognitive-motor trajectories	Higher concentrations associated with dual cognitive-motor decline and isolated motor decline	Significant	MMSE	Associated with cognitive decline
		p-tau181	0.94 [0.64–1.36]; 1.35 [0.79–1.87]; 1.47 [1.00–1.95]; 1.69 [1.14–2.57]	< 0.001	Gait	Cognitive-motor trajectories	Higher concentrations associated with dual cognitive-motor decline	Significant	MMSE	Associated with cognitive decline
		t-tau	0.75 [0.50–1.08]; 0.82 [0.58–1.16]; 0.89 [0.63–1.19]; 0.94 [0.63–1.23]	< 0.001	Gait	Cognitive-motor trajectories	Associated mainly with cognitive decline trajectories	Significant	MMSE	Associated with cognitive decline
		NfL	14.03 [10.77–18.59]; 23.17 [16.46–31.24]; 23.38 [16.48–35.45]; 29.94 [22.45–42.05]	< 0.001	Gait	Cognitive-motor trajectories	Higher concentrations associated with dual cognitive-motor decline	Significant	MMSE	Associated with cognitive decline
		GFAP	95.93 [66.52–136.93]; 146.20 [100.43–208.91]; 160.99 [110.99–266.41]; 203.98 [140.55–272.10]	< 0.001	Gait	Cognitive-motor trajectories	Associated primarily with cognitive decline trajectories	Significant	MMSE	Associated with cognitive decline

*AD* Alzheimer’s disease, *MCI* mild cognitive impairment, *NC* normal cognition, *CSF* cerebrospinal fluid, *MMSE* Mini-Mental State Examination, *SPPB* Short Physical Performance Battery, *TUG* Timed Up and Go, *NfL* neurofilament light chain, *GFAP* glial fibrillary acidic protein, *PGRN* progranulin, *Aβ42/40* amyloid-beta 42/40 ratio, *p-tau181* phosphorylated tau 181, *p-tau217* phosphorylated tau 217, *NR* not reported, *NS* not significant

Note: In Pinardi et al. (2026), motor function was operationalized using longitudinal gait-speed trajectories over a 15-year follow-up period. Participants were classified as slow/non-decliners, fast motor decliners, fast cognitive decliners, or dual cognitive-motor decliners

The most frequently investigated neurodegenerative biomarkers were Aβ42 [14, 15, 17, 19–22, 25, 26, 28], p-tau181 [14, 24, 29], total tau (t-tau) [13–15, 17, 20, 21, 25–28], phosphorylated tau (p-tau) [18, 20, 26], neurofilament light chain (NfL) [27, 29] brain-derived neurotrophic factor (BDNF) [22], Aβ40 [15, 17, 22], Aβ42/Aβ40 ratio [13, 16, 27], GFAP [27] and AB42/P-tau ratio [25]. Inflammatory biomarkers included TNF-α, IL-1β, IL-6, IL-8, IL-15, IGF-1, IGF-2, VEGF, FGF-2, CRP, and progranulin (PGRN) [16].

Studies reporting Aβ42 levels consistently found substantially higher concentrations in CSF than in blood-derived samples. In studies including both AD and MCI participants, lower CSF Aβ42 concentrations were generally observed in AD, reflecting increased cerebral amyloid deposition. Similarly, t-tau concentrations were markedly higher in CSF compared with plasma or serum samples, and studies including AD, MCI, and cognitively normal participants generally reported higher tau levels among individuals with AD. Only two studies reported directly comparable p-tau concentrations across diagnostic groups, showing higher levels among participants with cognitive impairment than cognitively normal controls. More recent studies additionally demonstrated elevated concentrations of p-tau181, p-tau217, NfL, and GFAP among individuals with accelerated cognitive and motor decline. Due to heterogeneity in study populations, biological matrices, and laboratory methods, direct quantitative comparisons across studies were limited.

### Motor function assessment and biomarkers

#### Alzheimer’s disease

Among participants with Alzheimer’s disease, neurodegenerative biomarkers were consistently associated with gait and mobility impairments. Lower CSF Aβ42 concentrations were associated with slower gait speed and poorer mobility performance measured using the Timed Up and Go (TUG) test [14, 25]. Higher CSF t-tau and p-tau concentrations were associated with impaired dual-task mobility and longer TUG completion times [14, 18, 20, 25]. Chen et al. [28] reported poorer gait performance among individuals with mild Alzheimer’s disease than cognitively normal participants; however, significant associations between plasma biomarkers and motor outcomes were primarily observed in the motoric cognitive risk syndrome group.

#### Mild cognitive impairment

Among participants with mild cognitive impairment, neurodegenerative biomarkers showed the most consistent associations with motor impairment. Lower CSF Aβ42 concentrations were associated with poorer gait and mobility performance, including slower gait speed, longer walking time, poorer dynamic balance, and impaired TUG performance [14, 18, 20, 25, 26]. Higher concentrations of tau biomarkers were also consistently associated with motor dysfunction. Increased CSF t-tau and p-tau levels were associated with poorer dual-task mobility and longer TUG completion times [18, 20, 25], while lower handgrip strength was associated with higher CSF tau and p-tau concentrations [26]. Overall, neurodegenerative biomarkers, particularly Aβ42, t-tau, and p-tau, demonstrated consistent associations with gait, mobility, balance, and muscle strength among individuals with MCI.

#### Cognitively unimpaired / healthy older adults

Among cognitively unimpaired older adults, blood-based neurodegenerative biomarkers were consistently associated with motor performance, particularly in longitudinal cohort studies. Plasma Aβ40, Aβ42, t-tau, and NfL were associated with gait speed, TUG performance, grip strength, and physical function assessed using the Short Physical Performance Battery (SPPB) [13, 15, 17, 27, 29]. Longitudinal evidence further demonstrated that higher plasma concentrations of p-tau181, p-tau217, NfL, and glial fibrillary acidic protein (GFAP) were associated with accelerated motor decline trajectories [27], whereas higher plasma p-tau181 and NfL concentrations were associated with poorer gait speed, balance, and expanded SPPB (eSPPB) performance [29]. Among inflammatory biomarkers, only serum IL-8 was significantly associated with mobility performance, while IL-6, TNF-α, IL-15, CRP, and the remaining inflammatory biomarkers showed no significant associations with motor outcomes [22, 23]. In addition, serum BDNF, Aβ40, and Aβ42 concentrations were positively associated with cardiorespiratory fitness (VO₂max) [22].

#### Adults with cognitive complaints and motoric cognitive risk syndrome

Among adults with cognitive complaints and those with MCR, blood-based neurodegenerative biomarkers were primarily associated with mobility and physical function. In participants with MCR, higher plasma total tau concentrations were associated with poorer cognitive and motor performance, while plasma Aβ42 concentrations were associated with gait and mobility impairment [21, 28]. In adults with cognitive complaints, higher plasma NfL concentrations were associated with lower SPPB scores, whereas higher PGRN concentrations predicted greater declines in handgrip strength over time [16]. Similarly, in the MEMENTO cohort, higher plasma p-tau181 and NfL concentrations were associated with poorer physical performance measured by the SPPB [24]. Overall, neurodegenerative biomarkers were more consistently associated with mobility and physical performance than inflammatory biomarkers in this population.

#### Cognitive function assessment

Eleven studies assessed cognitive function using the Mini-Mental State Examination (MMSE) or other standardized cognitive measures. Among participants with Alzheimer’s disease and mild cognitive impairment, tau-related biomarkers demonstrated the most consistent associations with cognitive impairment. Higher cerebrospinal fluid or plasma concentrations of t-tau, p-tau, and p-tau181 were associated with poorer cognitive performance and greater disease severity [21, 28]. Lower cerebrospinal fluid Aβ42 concentrations were generally observed in participants with Alzheimer’s disease compared with those with mild cognitive impairment and cognitively unimpaired individuals; however, significant associations between Aβ42 concentrations and cognitive test performance were inconsistent across studies [14, 21, 26].

Among cognitively unimpaired older adults and adults with cognitive complaints, longitudinal studies demonstrated that higher plasma NfL, p-tau181, p-tau217, and GFAP concentrations were associated with accelerated cognitive decline and dual cognitive-motor decline trajectories [24, 27]. In contrast, inflammatory biomarkers demonstrated limited associations with cognitive outcomes. Tsai et al. [22] found no significant relationship between inflammatory biomarkers and cognitive performance, while Legdeur et al. [23] reported no association between serum CRP concentrations and cognition. Overall, tau-related biomarkers, particularly p-tau181, p-tau217, t-tau, and NfL, demonstrated the strongest and most consistent associations with cognitive impairment across the Alzheimer’s disease continuum.

## Discussion

### Summary of the main findings

This systematic review indicates that cerebrospinal fluid and blood biomarkers related to Alzheimer’s disease are associated with motor function decline in individuals with AD, mild cognitive impairment, motoric cognitive risk syndrome, cognitive complaints, and cognitively unimpaired adults at risk of dementia. Across the 17 included studies, tau-related biomarkers, Aβ42, and NfL showed the most consistent associations with motor outcomes, particularly gait speed, mobility, balance, physical performance, and strength. Recent longitudinal evidence further demonstrated that elevated concentrations of p-tau181, p-tau217, NfL, and GFAP are associated with accelerated motor decline and dual cognitive-motor decline trajectories. In contrast, evidence regarding inflammatory biomarkers remains limited, with only isolated associations reported, primarily involving IL-8 and mobility performance.

### Contrast with literature

Among individuals with AD and MCI, gait impairment was the motor domain most consistently associated with biomarkers of neurodegeneration. Gait decline is one of the earliest motor manifestations of Alzheimer’s disease and is characterized by reduced walking speed, decreased cadence, prolonged double-support time, and increased gait variability [30, 31]. Walking requires the integration of multiple cortical and subcortical motor networks, including frontal, parietal, cerebellar, basal ganglia, and sensorimotor regions. Previous neuroimaging studies have demonstrated increased amyloid deposition in motor-related brain regions among cognitively normal individuals and patients with cognitive impairment [32, 33]. Similarly, the studies included in this review consistently demonstrated that lower CSF Aβ42 concentrations were associated with slower gait speed, whereas plasma Aβ42 showed less consistent associations. More recently, Ali et al. demonstrated that higher plasma concentrations of NfL, GFAP, and p-tau181 were independently associated with poorer gait performance in a large population-based cohort, supporting the concept that blood-based biomarkers reflect motor decline independently of cognitive status [34].

Mobility impairment and reduced physical performance also appear to emerge early during the Alzheimer’s disease continuum [4]. Performance on the TUG test has consistently differentiated cognitively healthy older adults from individuals with MCI and has shown moderate-to-strong associations with executive and frontal cognitive functions [35]. In the present review, amyloid-β and tau biomarkers demonstrated the strongest associations with TUG performance, whereas NfL was more consistently associated with SPPB scores. These findings agree with recent evidence demonstrating that blood biomarkers of neurodegeneration correlate not only with cognitive decline but also with functional motor impairment in aging populations [34].

Reduced muscle strength is another frequent finding throughout Alzheimer’s disease continuum. Lower handgrip strength has been associated with an increased risk of cognitive decline and dementia [36, 37] and patients with AD and MCI consistently exhibit lower strength than cognitively healthy individuals [38]. Sarcopenia has been proposed as one of the biological mechanisms linking motor dysfunction with neurodegeneration through alterations in myokines, neurotrophic factors, and inflammatory mediators such as IGF-1, IL-6, IL-8, IL-15, and BDNF [39]. Increased IL-6 and IL-8 concentrations have been associated with poorer cognitive performance [40], although their relationship with Alzheimer’s pathology remains inconsistent. Capogna et al. suggested that elevated IL-6 and IL-8 concentrations may even exert protective effects in individuals with lower AD pathology [41]. whereas other studies reported inverse associations with MMSE performance [42]. In contrast, evidence linking inflammatory biomarkers with motor performance remains scarce. Only one study included in this review demonstrated an association between serum IL-8 concentrations and 8-Foot Up-and-Go performance, indicating that additional longitudinal studies are required to clarify the contribution of peripheral inflammation to motor decline.

An important finding of this review is that significant associations between biomarkers and motor performance were also observed among cognitively unimpaired older adults and individuals with subjective cognitive complaints or motoric cognitive risk syndrome. Longitudinal studies demonstrated that elevated plasma concentrations of p-tau181, p-tau217, NfL, and GFAP were associated with accelerated motor decline years before dementia diagnosis, suggesting that motor dysfunction may represent an early clinical manifestation of Alzheimer’s disease pathology. These observations are supported by recent longitudinal studies showing that motor assessments improve prediction models based on blood biomarkers and may facilitate the early identification of individuals at increased risk for neurodegeneration [43].

Neuropathologically, Alzheimer’s disease is characterized by extracellular accumulation of amyloid-β plaques, intracellular aggregation of phosphorylated tau, and progressive neuroinflammation leading to neuronal dysfunction and death [44]. Damage to neural circuits results not only in cognitive impairment but also in motor dysfunction, which may precede cognitive decline [45]. Motor function depends on the integrity of widespread neural circuits integrating cortical, subcortical, cerebellar, and sensory pathways [46]. Damage involving the basal ganglia, thalamus, amygdala, and frontal motor networks has been associated with motor dysfunction in dementia [47]. Guo et al. demonstrated that poorer physical performance was associated with greater cerebral amyloid deposition, particularly within the lateral parietal, temporal, posterior cingulate, frontal, occipital, and precuneus cortices [48]. More recently, Gupta et al. demonstrated that greater amyloid, tau, and neurodegeneration burden within the primary motor cortex were associated with poorer dexterity and slower walking speed, providing direct evidence that Alzheimer’s pathology affects cortical motor networks [49]. Furthermore, Na et al. reported that regional tau accumulation within sensorimotor and frontoparietal cortices was independently associated with motor dysfunction, indicating that tau pathology contributes directly to motor impairment through both cognition-dependent and cognition-independent mechanisms [50].

Evidence from animal models further supports these observations. Amyloid-β accumulation has been identified not only in the brain but also in skeletal muscle and spinal cord, where it has been associated with demyelination, neuromuscular junction degeneration, sarcopenia, and impaired muscle contractility [51, 52]. Similarly, experimental models expressing mutant tau demonstrate early motor deficits accompanied by extensive spinal tau pathology [53]. In addition, cholinergic dysfunction, a hallmark of Alzheimer’s disease, may impair neuromuscular transmission and contribute to progressive motor decline [54].

### Strengths and limitations

This review has several strengths. It included a comprehensive and updated systematic search across multiple databases, incorporated recently published evidence, evaluated both neurodegenerative and inflammatory biomarkers, and included participants across the Alzheimer’s disease continuum, including cognitively unimpaired adults, individuals with subjective cognitive complaints, motoric cognitive risk syndrome, MCI, and AD. The inclusion of both cross-sectional and longitudinal studies allowed a broader understanding of the temporal relationship between biomarkers and motor decline.

Several limitations should also be acknowledged. First, relatively few studies specifically evaluated inflammatory biomarkers in relation to motor outcomes, limiting definitive conclusions regarding their clinical relevance. Second, substantial heterogeneity existed across studies regarding participant characteristics, biological samples (CSF, plasma, serum), laboratory techniques, biomarker panels, and motor assessment protocols, which precluded quantitative meta-analysis. Finally, most available evidence remains observational, preventing causal inference regarding the relationship between biomarker changes and motor decline.

### Importance for public health

Understanding the relationship between biomarkers and motor function has important implications for early detection, disease monitoring, and intervention strategies. Identifying biomarkers associated with physical dysfunction may improve early recognition of Alzheimer’s disease and facilitate cost-effective screening strategies for individuals at increased risk of dementia. Recent evidence also suggests that combining simple motor assessments with blood-based biomarkers substantially improves prediction of future neurodegeneration, supporting their incorporation into preventive screening programs [43]. Future studies should prioritize standardized biomarker panels, harmonized motor assessment protocols, and longer longitudinal follow-up to determine whether motor impairment consistently precedes cognitive decline. Larger prospective studies including individuals with subjective cognitive complaints and motoric cognitive risk syndrome are particularly needed to clarify the role of motor dysfunction as an early clinical marker of Alzheimer’s disease.

## Conclusions

This systematic review demonstrates that neurodegenerative biomarkers are consistently associated with motor dysfunction across the Alzheimer’s disease continuum, including cognitively unimpaired adults at risk, individuals with subjective cognitive complaints, MCR, MCI, and AD. Tau-related biomarkers, Aβ42, NfL, and more recently GFAP showed the strongest associations with gait, mobility, balance, and physical performance. Evidence regarding inflammatory biomarkers remains limited and heterogeneous, highlighting the need for additional longitudinal studies. Improved understanding of these biomarker–motor relationships may facilitate earlier diagnosis, better disease monitoring, and the development of targeted interventions aimed at preserving mobility and quality of life in individuals at risk of Alzheimer’s disease.

## Supplementary Information

Below is the link to the electronic supplementary material.

Supplementary Material 1

## References

[1] Lee J (2023) Mild cognitive impairment in relation to Alzheimer’s disease: an investigation of principles, classifications, ethics, and problems. Neuroethics 16(2):16

[2] Murphy MP, LeVine H (2010) 3rd. Alzheimer’s disease and the amyloid-beta peptide. J Alzheimers Dis 19(1):311–323 20061647 10.3233/JAD-2010-1221 PMC2813509

[3] Dubois B, von Arnim CAF, Burnie N, Bozeat S, Cummings J (2023) Biomarkers in Alzheimer’s disease: role in early and differential diagnosis and recognition of atypical variants. Alzheimers Res Ther 15(1):175 37833762 10.1186/s13195-023-01314-6 PMC10571241

[4] Buchman AS, Bennett DA (2011) Loss of motor function in preclinical Alzheimer’s disease. Expert Rev Neurother 11(5):665–676 21539487 10.1586/ern.11.57 PMC3121966

[5] Gras LZ, Kanaan SF, McDowd JM, Colgrove YM, Burns J, Pohl PS (2015) Balance and gait of adults with very mild Alzheimer disease. J Geriatr Phys Ther 38(1):1–7 24755691 10.1519/JPT.0000000000000020 PMC4632639

[6] de Paula JJ, Albuquerque MR, Lage GM, Bicalho MA, Romano-Silva MA, Malloy-Diniz LF (2016) Impairment of fine motor dexterity in mild cognitive impairment and Alzheimer’s disease dementia: association with activities of daily living. Braz J Psychiatry 38(3):235–238 27508398 10.1590/1516-4446-2015-1874 PMC7194270

[7] Palma GCS, Freitas TB, Bonuzzi GMG, Torriani-Pasin C (2023) Does cognitive impairment impact motor learning? a scoping review of elderly individuals with Alzheimer’s disease and mild cognitive impairment. Percept Mot Skills 130(5):1924–1951 37337358 10.1177/00315125231182732

[8] Ramírez F, Gutiérrez M (2021) Dual-task gait as a predictive tool for cognitive impairment in older adults: a systematic review. Front Aging Neurosci 1313:769462. eCollection 2021. 10.3389/fnagi.2021.769462 10.3389/fnagi.2021.769462 PMC8740025 35002676

[9] Ogawa Y, Kaneko Y, Sato T, Shimizu S, Kanetaka H, Hanyu H (2018) Sarcopenia and muscle functions at various stages of Alzheimer disease. Front Neurol 9:9:710. eCollection 2018. 10.3389/fneur.2018.00710 10.3389/fneur.2018.00710 PMC6121095 30210435

[10] Andrade-Guerrero J, Martínez-Orozco H, Villegas-Rojas MM, Santiago-Balmaseda A, Delgado-Minjares KM, Pérez-Segura I et al (2024) Alzheimer’s disease: understanding motor impairments. Brain Sci 14(11):1054. 10.3390/brainsci14111054 10.3390/brainsci14111054 PMC11592238 39595817

[11] Kinney JW, Bemiller SM, Murtishaw AS, Leisgang AM, Salazar AM, Lamb BT (2018) Inflammation as a central mechanism in Alzheimer’s disease. Alzheimers Dement (N Y) 4:575–590 30406177 10.1016/j.trci.2018.06.014 PMC6214864

[12] Gunes S, Aizawa Y, Sugashi T, Sugimoto M, Rodrigues PP (2022) Biomarkers for Alzheimer’s disease in the current state: a narrative review. Int J Mol Sci 23(9):4962. 10.3390/ijms23094962 10.3390/ijms23094962 PMC9102515 35563350

[13] Paulsen AJ, Schubert CR, Pinto AA, Chappell RJ, Chen Y, Cruickshanks KJ et al (2022) Associations of sensory and motor function with blood-based biomarkers of neurodegeneration and Alzheimer’s disease in midlife. Neurobiol Aging 120:177–188 36209638 10.1016/j.neurobiolaging.2022.08.008 PMC9613601

[14] Tangen GG, Sverdrup K, Taraldsen K, Persson K, Engedal K, Bekkhus-Wetterberg P et al (2023) Mobility and associations with levels of cerebrospinal fluid amyloid β and tau in a memory clinic cohort. Front Aging Neurosci 15:1101306 36820757 10.3389/fnagi.2023.1101306 PMC9939466

[15] Jacob ME, O’Donnell A, Samra J, Gonzales MM, Satizabal C, Pase MP et al (2022) Grip strength, gait speed and plasma markers of neurodegeneration in asymptomatic middle-aged and older adults. J Frailty Aging 11(3):291–298 35799435 10.14283/jfa.2022.17

[16] He L, de Souto Barreto P, Giudici KV, Aggarwal G, Nguyen AD, Morley JE et al (2021) Cross-sectional and longitudinal associations between plasma neurodegenerative biomarkers and physical performance among community-dwelling older adults. J Gerontol Biol Sci Med Sci 76(10):1874–1881 10.1093/gerona/glaa284 33186456

[17] O’Bryant SE, Petersen M, Hall JR, Large S, Johnson LA (2023) Plasma biomarkers of Alzheimer’s disease are associated with physical functioning outcomes among cognitively normal adults in the multiethnic HABS-HD cohort. J Gerontol Biol Sci Med Sci 78(1):9–15 10.1093/gerona/glac169 PMC9879752 35980599

[18] Nilsson MH, Tangen GG, Palmqvist S, van Westen D, Mattsson-Carlgren N, Stomrud E et al (2021) The effects of tau, amyloid, and white matter lesions on mobility, dual tasking, and balance in older people. J Gerontol Biol Sci Med Sci 76(4):683–691 10.1093/gerona/glaa143 PMC8011701 32506119

[19] Skillbäck T, Blennow K, Zetterberg H, Skoog J, Rydén L, Wetterberg H et al (2022) Slowing gait speed precedes cognitive decline by several years. Alzheimers Dement 18(9):1667–1676 35142034 10.1002/alz.12537 PMC9514316

[20] Åhman HB, Giedraitis V, Cedervall Y, Lennhed B, Berglund L, McKee K et al (2019) Dual-task performance and neurodegeneration: correlations between timed up-and-go dual-task test outcomes and Alzheimer’s disease cerebrospinal fluid biomarkers. J Alzheimers Dis 71(s1):S75–s83 31104024 10.3233/JAD-181265 PMC6839487

[21] Chen P-H, Lin S-I, Liao Y-Y, Hsu W-L, Cheng F-Y (2022) Associations between blood-based biomarkers of Alzheimer’s disease with cognition in motoric cognitive risk syndrome: A pilot study using plasma Aβ42 and total tau. Front Aging Neurosci 14:1125201. 10.3389/fnagi.2022.1125201 10.3389/fnagi.2022.1125201 PMC9850215 36688149

[22] Tsai CL, Erickson KI, Sun HS, Kuo YM, Pai MC (2021) A cross-sectional examination of a family history of Alzheimer’s disease and ApoE epsilon 4 on physical fitness, molecular biomarkers, and neurocognitive performance. Physiol Behav 230:113268 33383402 10.1016/j.physbeh.2020.113268

[23] Legdeur N, Badissi M, Yaqub M, Beker N, Sudre CH, Ten Kate M et al (2021) What determines cognitive functioning in the oldest-old? The EMIF-AD 90 + study. J Gerontol B Psychol Sci Soc Sci 76(8):1499–1511 32898275 10.1093/geronb/gbaa152

[24] Grasset L, Bouteloup V, Cacciamani F, Pellegrin I, Planche V, Chêne G et al (2024) Associations between blood-based biomarkers and cognitive and functional trajectories among participants of the MEMENTO cohort. Neurology 102(9):e209307 38626384 10.1212/WNL.0000000000209307 PMC11175638

[25] Nielsen MS, Simonsen AH, Siersma V, Hasselbalch SG, Hoegh P (2018) The diagnostic and prognostic value of a dual-tasking paradigm in a memory clinic. J Alzheimers Dis 61(3):1189–1199 29278887 10.3233/JAD-161310

[26] Sampatakakis SN, Mamalaki E, Ntanasi E, Kalligerou F, Liampas I, Yannakoulia M et al (2023) Objective physical function in the Alzheimer’s disease continuum: association with cerebrospinal fluid biomarkers in the ALBION study. Int J Mol Sci [Internet] 24(18) :14079. 10.3390/ijms241814079 10.3390/ijms241814079 PMC10531412 37762384

[27] Pinardi E, Grande G, Ornago AM, Valletta M, Rizzuto D, Fredolini C et al (2026) Blood biomarkers of Alzheimer’s disease and 15-year decline in cognitive and motor functions in older adults. J Intern Med 300(1):64–77 42121219 10.1111/joim.70110

[28] Chen PH, Lin SI, Liao YY, Hu GC, Cheng FY (2025) Plasma Alzheimer’s biomarkers and physical functions in aging adults with and without motoric cognitive risk syndrome. Exp Gerontol 212:112975 41297721 10.1016/j.exger.2025.112975

[29] Thompson AC, Leng X, Miller ME, Register TC, Laurienti PJ, Mielke MM et al (2025) Relationship of Alzheimer’s disease and related dementias plasma biomarkers with mobility in cognitively unimpaired older adults. J Gerontol Biol Sci Med Sci 80(7) :glaf110. 10.1093/gerona/glaf110 10.1093/gerona/glaf110 PMC12198666 40378095

[30] Dumurgier J, Artaud F, Touraine C, Rouaud O, Tavernier B, Dufouil C et al (2017) Gait speed and decline in gait speed as predictors of incident dementia. J Gerontol Biol Sci Med Sci 72(5):655–661 10.1093/gerona/glw110 27302701

[31] Nadkarni NK, Mawji E, McIlroy WE, Black SE (2009) Spatial and temporal gait parameters in Alzheimer’s disease and aging. Gait Posture 30(4):452–454 19740661 10.1016/j.gaitpost.2009.07.003 PMC4030705

[32] Wennberg AMV, Savica R, Hagen CE, Roberts RO, Knopman DS, Hollman JH et al (2017) Cerebral amyloid deposition is associated with gait parameters in the mayo clinic study of aging. J Am Geriatr Soc 65(4):792–799 27869301 10.1111/jgs.14670 PMC5397339

[33] Dao E, Hsiung G-YR, Sossi V, Tam R, Shahinfard E, Nicklin E et al (2019) Cerebral amyloid-β deposition is associated with impaired gait speed and lower extremity function. J Alzheimer’s Disease 71(s1):S41–S9 30741682 10.3233/JAD-180848

[34] Ali F, Syrjanen JA, Figdore DJ, Kremers WK, Mielke MM, Jack CR et al (2025) Association of plasma biomarkers of Alzheimer’s pathology and neurodegeneration with gait performance in older adults. Commun Med (Lond) 5(1):19 39820537 10.1038/s43856-024-00713-6 PMC11739691

[35] Melo LM, Ansai JH, Ferreira ACVG, Silva DCP, Vale FAC, Takahashi ACM et al (2022) Correlation between changes in timed up and go performance and cognition in older people with mild cognitive impairment: A longitudinal study. Clin Biomech Elsevier Ltd 94:105620 10.1016/j.clinbiomech.2022.105620 35325714

[36] Cui M, Zhang S, Liu Y, Gang X, Wang G (2021) Grip strength and the risk of cognitive decline and dementia: a systematic review and meta-analysis of longitudinal cohort studies. Front Aging Neurosci 13:625551 33613270 10.3389/fnagi.2021.625551 PMC7890203

[37] Duchowny KA, Ackley SF, Brenowitz WD, Wang J, Zimmerman SC, Caunca MR et al (2022) Associations between handgrip strength and dementia risk, cognition, and neuroimaging outcomes in the UK biobank cohort study. JAMA Netw Open 5(6):e2218314–e 35737388 10.1001/jamanetworkopen.2022.18314 PMC9227006

[38] Kuo K, Zhang YR, Chen SD, He XY, Huang SY, Wu BS et al (2023) Associations of grip strength, walking pace, and the risk of incident dementia: A prospective cohort study of 340212 participants. Alzheimers Dement 19(4):1415–1427 36152312 10.1002/alz.12793

[39] Han X, Ashraf M, Tipparaju SM, Xuan W (2023) Muscle–brain crosstalk in cognitive impairment. Front Aging Neurosci 15:1221653. 10.3389/fnagi.2023.1221653 10.3389/fnagi.2023.1221653 PMC10413125 37577356

[40] Teoh NSN, Gyanwali B, Lai MKP, Chai YL, Chong JR, Chong EJY et al (2023) Association of Interleukin-6 and Interleukin-8 with Cognitive Decline in an Asian Memory Clinic Population. J Alzheimers Dis 92(2):445–455 36776060 10.3233/JAD-220971

[41] Capogna E, Watne LO, Sørensen Ø, Guichelaar CJ, Idland AV, Halaas NB et al (2023) Associations of neuroinflammatory IL-6 and IL-8 with brain atrophy, memory decline, and core AD biomarkers – in cognitively unimpaired older adults. Brain Behav Immun 113:56–65 37400002 10.1016/j.bbi.2023.06.027

[42] Lyra e Silva NM, Gonçalves RA, Pascoal TA, Lima-Filho RAS, Resende EPF, Vieira ELM et al (2021) Pro-inflammatory interleukin-6 signaling links cognitive impairments and peripheral metabolic alterations in Alzheimer’s disease. Translational Psychiatry 11(1):251 33911072 10.1038/s41398-021-01349-z PMC8080782

[43] Paulsen AJ, Pinto AA, Schubert CR, Chappell RJ, Chen Y, Engelman CD et al (2024) Midlife sensory and motor functions improve prediction of blood-based measures of neurodegeneration and Alzheimer’s disease in late middle-age. Alzheimers Dement (Amst) 16(1):e12564 38476637 10.1002/dad2.12564 PMC10927920

[44] Trejo-Lopez JA, Yachnis AT, Prokop S (2022) Neuropathology of Alzheimer’s disease. Neurotherapeutics 19(1):173–185 34729690 10.1007/s13311-021-01146-y PMC9130398

[45] Devanand DP, Liu X, Tabert MH, Pradhaban G, Cuasay K, Bell K et al (2008) Combining early markers strongly predicts conversion from mild cognitive impairment to Alzheimer’s disease. Biol Psychiatry 64(10):871–879 18723162 10.1016/j.biopsych.2008.06.020 PMC2613777

[46] Svoboda K, Li N (2018) Neural mechanisms of movement planning: motor cortex and beyond. Curr Opin Neurobiol 49:33–41 29172091 10.1016/j.conb.2017.10.023

[47] Schirinzi T, Di Lorenzo F, Sancesario GM, Di Lazzaro G, Ponzo V, Pisani A et al (2018) Amyloid-mediated cholinergic dysfunction in motor impairment related to Alzheimer’s disease. J Alzheimer’s Disease 64(2):525–532 29914023 10.3233/JAD-171166

[48] Guo Y, Huang L, Kuang J, Sun T, Zhang X, Tian H et al (2024) Physical function is associated with cognitive status, brain amyloid-beta deposition, and blood biomarkers in Chinese Han population. CNS Neurosci Ther 30(8):e14921 39155519 10.1111/cns.14921 PMC11330986

[49] Gupta L, Ma Y, Kohli A, Yang KL, Oh JM, Betthauser TJ et al (2024) Alzheimer’s disease biomarker burden in primary motor cortices is associated with poorer dexterity performance. Alzheimers Dement 20(8):5792–5799 38934641 10.1002/alz.13899 PMC11350021

[50] Na HK, Cho H, Lee HS, Kim HK, Yoon S, Lee JH et al (2025) Neural basis of motor symptoms in Alzheimer’s disease: role of regional tau burden and cognition. Alzheimers Dement 21(8):e70598 40810249 10.1002/alz.70598 PMC12351292

[51] Yuan Q, Yang J, Wu W, Lin ZX (2017) Motor deficits are independent of axonopathy in an Alzheimer’s disease mouse model of TgCRND8 mice. Oncotarget 8(58):97900–97912 29228660 10.18632/oncotarget.18429 PMC5716700

[52] Oveisgharan S, Wang T, Barnes LL, Schneider JA, Bennett DA, Buchman AS (2024) The time course of motor and cognitive decline in older adults and their associations with brain pathologies: a multicohort study. Lancet Healthy Longev 5(5):e336–e45 38582095 10.1016/S2666-7568(24)00033-3 PMC11129202

[53] Yoshiyama Y, Higuchi M, Zhang B, Huang SM, Iwata N, Saido TC et al (2007) Synapse loss and microglial activation precede tangles in a P301S tauopathy mouse model. Neuron 53(3):337–351 17270732 10.1016/j.neuron.2007.01.010

[54] Monteiro-Cardoso VF, Castro M, Oliveira MM, Moreira PI, Peixoto F, Videira RA (2015) Age-dependent biochemical dysfunction in skeletal muscle of triple-transgenic mouse model of Alzheimer`s disease. Curr Alzheimer Res 12(2):100–115 25654504 10.2174/1567205012666150204124852 PMC4428479

