# Supplementary material for: Motor performance and its association with Alzheimer’s-related biomarkers: a systematic review
Source: Neurol Sci. 2026 Jul 31;47(8):672. doi: 10.1007/s10072-026-09253-4 (PMC13427869; doi:10.1007/s10072-026-09253-4)
Supplement: Supplementary file 1 — Supplementary Material 1 [file 10072_2026_9253_MOESM1_ESM.docx]

**Table S1.** Methodological quality assessment of cross-sectional studies using the adapted Newcastle–Ottawa Scale (NOS).

| **Study** | **Selection (5)** | **Comparability (2)** | **Outcome (2)** | **Total Score** | **Quality** |
| --- | --- | --- | --- | --- | --- |
| Åhman et al., 2019 | 4 | 0 | 2 | **6/9** | Moderate |
| Legdeur et al., 2020 | 4 | 1 | 2 | **7/9** | High |
| Nilsson et al., 2020 | 4 | 2 | 2 | **8/9** | High |
| Tsai et al., 2021 | 4 | 0 | 2 | **6/9** | Moderate |
| Chen et al., 2022 | 4 | 0 | 2 | **6/9** | Moderate |
| O'Bryant et al., 2023 | 5 | 2 | 2 | **9/9** | High |
| Sampatakakis et al., 2023 | 4 | 1 | 2 | **7/9** | High |
| Tangen et al., 2023 | 4 | 1 | 2 | **7/9** | High |
| Thompson et al., 2025 | 4 | 2 | 2 | **8/9** | High |
| Chen et al., 2025 | 4 | 1 | 2 | **7/9** | High |

**Table S2.** Methodological quality assessment of longitudinal studies using the Newcastle–Ottawa Scale (NOS).

| **Study** | **Selection (4)** | **Comparability (2)** | **Outcome (3)** | **Total Score** | **Quality** |
| --- | --- | --- | --- | --- | --- |
| Nielsen et al., 2018 | 3 | 1 | 2 | **6/9** | Moderate |
| He et al., 2020 | 3 | 1 | 2 | **6/9** | Moderate |
| Skillbäck et al., 2021 | 3 | 2 | 2 | **7/9** | High |
| Paulsen et al., 2022 | 4 | 2 | 2 | **8/9** | High |
| Jacob et al., 2022 | 3 | 2 | 2 | **7/9** | High |
| Grasset et al., 2024 | 4 | 2 | 2 | **8/9** | High |
| Pinardi et al., 2026 | 4 | 2 | 2 | **8/9** | High |

**Appendix S1.** Detailed search strategy used for each electronic database.

| **DATABASE** | **FULL SEARCH STRATEGY** |
| --- | --- |
| **MEDLINE (OVID)** | 1. exp Alzheimer Disease/ 2. exp Mild Cognitive Impairment/ 3. "motoric cognitive risk".mp. 4. "subjective cognitive decline".mp. 5. "cognitive complaints".mp. 6. "cognitive decline".mp. 7. 1 OR 2 OR 3 OR 4 OR 5 OR 6  8. exp Biomarkers/ 9. biomarker*.mp. 10. blood biomarker*.mp. 11. peripheral biomarker*.mp. 12. neurodegenerative biomarker*.mp. 13. inflammatory biomarker*.mp. 14. exp Inflammation/ 15. inflammat*.mp. 16. 8 OR 9 OR 10 OR 11 OR 12 OR 13 OR 14 OR 15  17. exp Motor Activity/ 18. exp Walking/ 19. exp Gait/ 20. gait.mp. 21. gait speed.mp. 22. walking speed.mp. 23. mobility.mp. 24. balance.mp. 25. motor function.mp. 26. motor performance.mp. 27. physical performance.mp. 28. physical function.mp. 29. 17 OR 18 OR 19 OR 20 OR 21 OR 22 OR 23 OR 24 OR 25 OR 26 OR 27 OR 28 |
| **Web of Science** | TS = (("Alzheimer disease" OR "Alzheimer's disease" OR "mild cognitive impairment" OR "motoric cognitive risk" OR "subjective cognitive decline" OR "cognitive complaints" OR "cognitive decline") AND (biomarker* OR "blood biomarker*" OR "peripheral biomarker*" OR "neurodegenerative biomarker*" OR "inflammatory biomarker*" OR inflammat*) AND ("motor function" OR "motor performance" OR "physical function" OR "physical performance" OR gait OR "gait speed" OR "walking speed" OR walking OR mobility OR balance)) |
| **SCOPUS** | TITLE-ABS-KEY (("Alzheimer disease" OR "Alzheimer's disease" OR "mild cognitive impairment" OR "motoric cognitive risk" OR "subjective cognitive decline" OR "cognitive complaints" OR "cognitive decline") AND (biomarker* OR "blood biomarker*" OR "peripheral biomarker*" OR "neurodegenerative biomarker*" OR "inflammatory biomarker*" OR inflammat*) AND ("motor function" OR "motor performance" OR "physical function" OR "physical performance" OR gait OR "gait speed" OR "walking speed" OR walking OR mobility OR balance)) |
| **LILACS** | ((tw:("Alzheimer disease" OR "Alzheimer's disease" OR "enfermedad de Alzheimer" OR "mild cognitive impairment" OR "deterioro cognitivo leve" OR "motoric cognitive risk" OR "subjective cognitive decline" OR "cognitive complaints" OR "declínio cognitivo"))) AND (tw:(biomarker OR biomarcador OR "blood biomarker*" OR "peripheral biomarker*" OR "neurodegenerative biomarker*" OR "inflammatory biomarker*" OR inflammat* OR inflam* OR inflamação)) AND (tw:("motor function" OR "motor performance" OR "physical function" OR "physical performance" OR gait OR marcha OR "walking speed" OR "gait speed" OR movilidad OR mobilidade OR balance OR equilíbrio))) |

Note: The MEDLINE strategy was adapted for the CENTRAL database using the same search concepts and database-specific indexing terms.
